# New Insights into the Diversity of Branchiomeric Muscle Development: Genetic Programs and Differentiation

**DOI:** 10.3390/biology11081245

**Published:** 2022-08-22

**Authors:** Imadeldin Yahya, Dorit Hockman, Beate Brand-Saberi, Gabriela Morosan-Puopolo

**Affiliations:** 1 Department of Anatomy and Molecular Embryology, Ruhr University Bochum, 44801 Bochum, Germany; 2 Department of Anatomy, Faculty of Veterinary Medicine, University of Khartoum, Khartoum 11115, Sudan; 3 Division of Cell Biology, Department of Human Biology, Neuroscience Institute, Faculty of Health Sciences, University of Cape Town, Cape Town 7700, South Africa

**Keywords:** branchiomeric muscles, differentiation, mouse embryo, neural crest cells

## Abstract

**Simple Summary:**

We review the transcription factors and signaling molecules driving differentiation of a subset of head muscles known as the branchiomeric muscles due to their origin in the pharyngeal arches. We provide novel data on the distinct myogenic programs within these muscles and explore how the cranial neural crest cell regulates branchiomeric muscle patterning and differentiation.

**Abstract:**

Branchiomeric skeletal muscles are a subset of head muscles originating from skeletal muscle progenitor cells in the mesodermal core of pharyngeal arches. These muscles are involved in facial expression, mastication, and function of the larynx and pharynx. Branchiomeric muscles have been the focus of many studies over the years due to their distinct developmental programs and common origin with the heart muscle. A prerequisite for investigating these muscles’ properties and therapeutic potential is understanding their genetic program and differentiation. In contrast to our understanding of how branchiomeric muscles are formed, less is known about their differentiation. This review focuses on the differentiation of branchiomeric muscles in mouse embryos. Furthermore, the relationship between branchiomeric muscle progenitor and neural crest cells in the pharyngeal arches of chicken embryos is also discussed. Additionally, we summarize recent studies into the genetic networks that distinguish between first arch-derived muscles and other pharyngeal arch muscles.

## 1. Introduction

During vertebrate gastrulation, the embryo differentiates into three germ layers: the endoderm, mesoderm, and ectoderm. Skeletal muscles predominately originate from the embryonic middle germ layer, the mesoderm. Although the structure and repair of all skeletal muscles are the same, head muscles differ from trunk muscles in several respects [1,2,3,4]. Head muscle progenitor cells originate at distinct embryonic locations. Differences in their gene regulatory networks and transcriptional mechanisms can also be noted [5,6,7,8]. The most remarkable feature of the head muscles is that their progenitor cells contribute to both types of striated muscles (skeletal and cardiac). In addition to these distinguishing differences, it should also be mentioned that their connective tissue derives from a different source than that of the trunk muscle. In this review, we have attempted to bring together much of the recent research on branchiomeric muscle’s origin and genetic program. We also intend to provide a critical overview of the relationship between neural crest cells and pharyngeal mesoderm during the development of branchiomeric muscles. Currently, most research on the differentiation of branchiomeric muscle is restricted to the avian model. Furthermore, research concerning the differentiation of avian craniofacial muscles has previously been reviewed [9,10]. Consequently, this review focuses on the differentiation of mouse branchiomeric muscles. We also summarize our recent findings on the emergence of myosin heavy chain (MyHC) expression during branchiomeric muscle development and the role of the ectomesenchyme neural crest cells in branchiomeric muscle development.

## 2. An Overview of the Early Trunk and Limb Muscle Development

The skeletal muscle of the trunk derives from the segmented paraxial mesoderm [11,12,13,14]. In response to secreted signals from surrounding tissues, the somites differentiate along the dorsal–ventral axis into a dorsal and a ventral segment [15,16,17]. Moreover, the ventral part undergoes an epithelial-to-mesenchymal transition (EMT) to form the sclerotome [12,13], which subsequently develops into the axial cartilage and bone of the vertebrae and ribs [12,16,18]. The dorsal segment of the somite retains its epithelial structure for longer and is known as the dermomyotome [5,12]. The dermomyotome is the source of the dorsal dermis, the skeletal muscles of the trunk and limbs, smooth muscle cells of blood vessels and endothelial, and brown fat [5]. Later in development, a third region forms when cells from the dorsomedial and ventrolateral lips of the dermomyotome delaminate and migrate to form the myotome [15]. The epaxial component of the myotome, which contributes to deep back muscles, develops from the dorsomedial lips of the dermomyotome. A similar pattern of events induces the ventrolateral lips to form the hypaxial component, which is the source of the ventrolateral body wall muscles and medial shoulder girdle muscles [12,16,19,20]. Cells from the ventrolateral dermomyotomal lips also undergo an EMT, delaminate, and migrate as single cells over long distances using stereotypic routes [12,16,21,22,23,24]. Consequently, these migrating progenitor cells generate the hypaxial muscles of the limbs, the lateral shoulder girdle diaphragm, and the tongue [12,17,21].

## 3. An Overview of Early Branchiomeric Muscle Development

**Figure 1:**
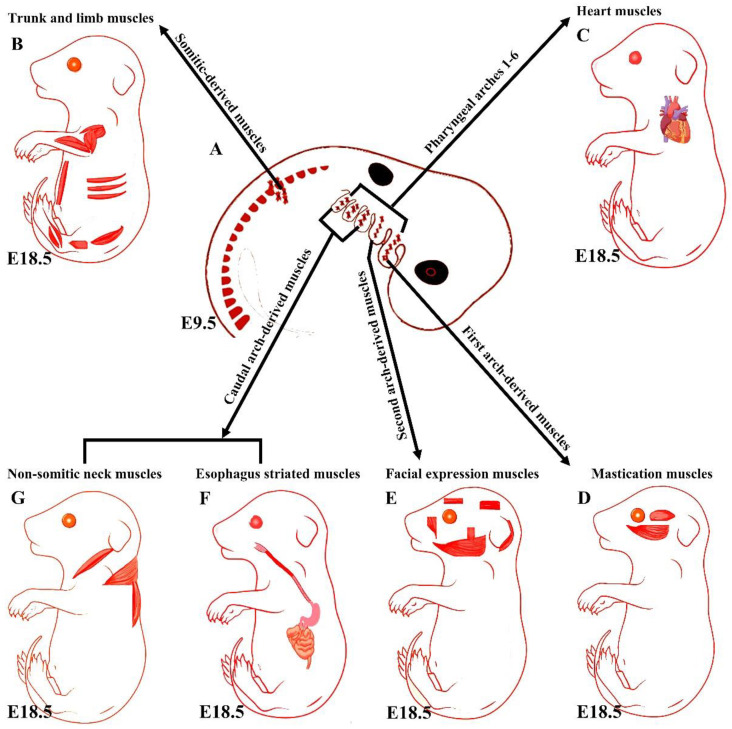
Summary of the embryonic origins of the branchiomeric and trunk muscles. (**A**) The branchiomeric muscle anlagen and second heart field progenitor cells originate from the cardiopharyngeal mesoderm that colonizes the core of the pharyngeal arches. (**B**) The somitic mesoderm gives rise to trunk and limb muscles. (**C**) The cardiopharyngeal mesoderm of arches 1–6 gives rise to cardiac muscle. (**D**) The cardiopharyngeal mesoderm of the first pharyngeal arch gives rise to mastication muscles. (**E**) The cardiopharyngeal mesoderm of the second pharyngeal arch gives rise to facial expression muscles. The caudal cardiopharyngeal mesoderm gives rise to the striated muscles of the esophagus (**F**) and non-somitic neck muscles (**G**). Retrieved from https://app.biorender.com/biorender-templates (accessed on 7 July 2022).

Although head muscles resemble limb and trunk muscles in myofiber architecture, their developmental history is widely divergent [11]. Branchiomeric muscles and their accompanying muscle stem cells develop from the cranial mesoderm (also known as pharyngeal mesoderm), which includes both the cranial paraxial mesoderm and lateral splanchnic mesoderm [1,25,26,27,28]. The pharyngeal mesoderm forms the mesodermal core within the pharyngeal arches (also known as branchial arches), which are transitory structures in the vertebrate embryo that bulge ventrally in pairs from the pharynx [1,5]. Each arch comprises a mesodermal core surrounded by neural crest cells, endoderm, and ectoderm, which tightly influence mesodermal cell development [26,29]. The mesodermal core of the pharyngeal arches gives rise to the branchiomeric muscles and significant parts of the heart [1,6,26,29,30,31,32,33,34]. The first and second pharyngeal arches give rise to masticatory and facial expression muscles, and posterior pharyngeal arches give rise to non-somitic neck muscles and esophagus striated muscles, respectively (Figure 1) [1,6,25,26,28,31,35]. Moreover, a recent mouse genetic lineage analysis revealed that pharyngeal mesoderm contributes to the medial pharyngeal skeleton and branchiomeric muscle elements (connective tissue) [36].

## 4. Distinct Genetic Programs in Branchiomeric Muscles

**Figure 2:**
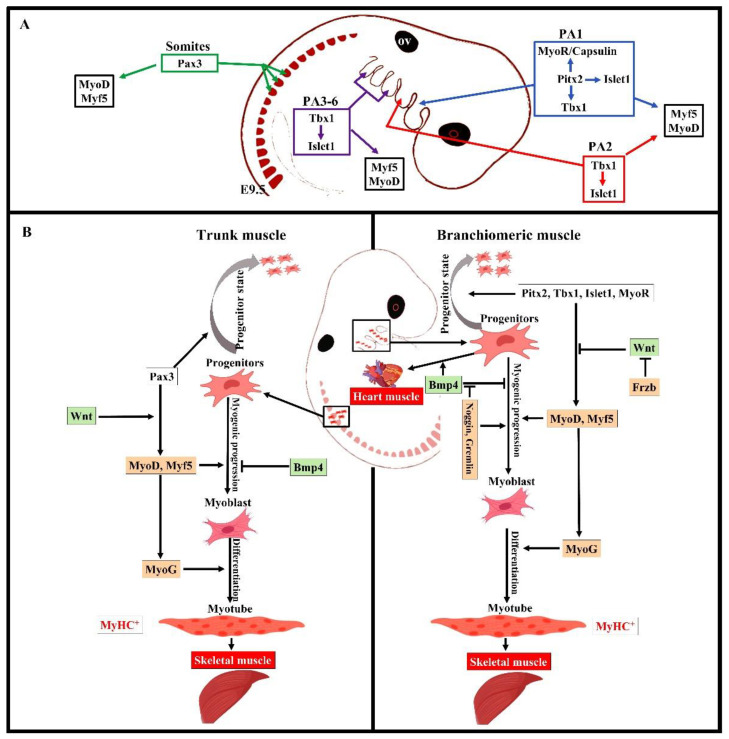
Summary of the distinct genetic program that governs myogenesis in the branchiomeric and trunk muscles. (**A**,**B**) Model of the genetic networks involved in branchiomeric and trunk muscles. The transcription factors Pitx2, Tbx1, Islet1, and MyoR set up the cardiopharyngeal mesoderm as a skeletal/heart-muscle-competent tissue. Pitx2 is required for the first pharyngeal arch muscle specification by modulating pre-myogenic markers (Tbx1, Capsulin, and MyoR). These genes are required for the activation of myogenic regulatory factors (MyoD and Myf5). The onset of Myf5 and MyoD commits branchiomeric muscle specification. MyoD directly activates genes implicated in keeping myoblasts in a proliferative state, whereas MyoG has antiproliferative activity through the activation of genes that block cell proliferation, promoting cell cycle exit and entry into terminal differentiation [45]. Pitx2 regulates the expression of Islet1, a second heart field marker. Tbx1 is required for the specification of second and caudal pharyngeal muscles. Tbx1 also regulates the expression of Islet1. Initiation of the myogenic program in the trunk and limb is regulated by Pax3, which is not expressed in the cardiopharyngeal mesoderm. BMP4 signals promote cardiogenesis in the head region and block skeletal muscle myogenesis in both the trunk and branchiomeric muscles. Wnt signaling inhibits branchiomeric muscle formation and initiates myogenesis in the trunk region. Antagonists of BMP4 (Noggin and Cremlin) and Wnt (Frzb) signals block cardiogenesis and induce the formation of branchiomeric muscle. PA, pharyngeal arch; ov, otic vesicle. Retrieved from https://app.biorender.com/biorender-templates (accessed on 7 July 2022).

Overall, the early stages of the myogenic progression can be followed by switching on the basic helix–loop–helix myogenic regulatory factors MyoD, Myf5, myogenin (MyoG), and MRF4 in all areas of the body [8] (Figure 2). In the trunk, Pax3 and Pax7 are expressed in the somites as soon as they form [2,12]. Pax3 keeps myogenic precursor cells in a proliferative state, but contributes to the onset of myogenesis and thus is referred to as a premyogenic gene [2]. In the somitic mesoderm, MyoD and Myf5 are expressed first, committing cells to myogenesis, and are therefore known as myogenic determination factors [2,12]. While trunk muscle progenitor cells require Pax3 expression for activating myogenic progression, branchiomeric muscle progenitor cells are regulated by a Pax3-independent regulatory network [8,25]. Branchiomeric muscles express a remarkably heterogeneous set of genes in both the embryo and adult. Molecular and technical advances in the last 20 years have provided comprehensive information about the genetic regulation of these muscles. Their myoblasts are specified by Pitx2, Tbx1, Islet1, musculin, and Capsulin genes ([7,8,14,25,37]). These genes also distinguish branchiomeric muscle satellite cells from satellite cells in the trunk [37,38,39]. Tbx1, Pitx2, and MyoR have been shown to maintain myogenic progenitor cells in an undifferentiated state, but are also required to initiate myogenesis similarly to Pax3 in the trunk [7,37,40,41,42,43,44]. Although all branchiomeric muscles share a common embryonic origin, the upstream factors involved in each pharyngeal arch are varied. In the mouse, Pitx2 is expressed in the mesodermal core of the first pharyngeal arch at E9.5. It acts to assure the expression of pre-myogenic genes Tbx1, Capsulin, and Musculin in the first arch-derived muscle, but not the second arch muscle [8]. Importantly, the first, but not second, arch mesoderm of Pitx2-null embryos failed to activate these transcription factors after E 9.5. Thus, Pitx2 is required to initiate the myogenic progression in the first arch mesoderm, but not in other pharyngeal arches [8,14]. The onset of myogenic progression in the second and most caudal pharyngeal arches is regulated by Tbx1, which regulates Myf5 and MyoD (Figure 2) [14,43]. In the absence of Tbx1, the caudal pharyngeal arches do not form, resulting in the absence of muscles developed from most caudal arches, including those of the larynx and esophagus [6,25,35]. Although Tbx1 is not required for the migration of the pharyngeal mesoderm into the first pharyngeal arch [43], it is required for the correct patterning of muscles with pharyngeal-mesoderm-derived connective tissue [36]. Previously, we reported on a fate-mapping experiment based on EGFP-based cell labeling and quail–chicken cell injection that found that chicken second pharyngeal arch progenitor cells contributed to the heart muscle in vivo [33]. We also reported that the chemokine receptor CXCR4 was required for the migration of pharyngeal mesoderm into the second and most caudal pharyngeal arches, but not the first pharyngeal arch. Interestingly, we also reported a reduction in muscles derived from the caudal pharyngeal arches (non-somitic neck muscle) in CXCR4 mutants [27,28]. Taken together, these findings suggested that the genetic programs promoting branchiomeric myogenesis in the various pharyngeal arches are widely divergent.

## 5. The Relationship between Branchiomeric Muscle Progenitors and Neural Crest Cells in Chicken Embryos

In vertebrates, the development of musculoskeletal systems requires an interdependent programming event. The morphogenesis of branchiomeric muscles necessitates tight integration with their surrounding connective tissue progenitor cells (cranial neural crest cells) [46,47,48,49]. The neural crest cells are a transient and multipotent progenitor cell population that emerges from the dorsal neural tube during early development [50,51]. Following induction, neural crest cells delaminate and migrate into the periphery to many sites at which they stop and differentiate into a broad range of cell types based on their axial level of origin [51,52,53]. They can be divided into four major subpopulations: cranial, cardiac, vagal, and trunk [54]. In the head region, neural crest cells can be grouped into cranial and cardiac neural crest cells [27,28,54]. Cardiac neural crest cells originating from the level of the hindbrain transit through the posterior pharyngeal arches before entering the heart and forming the aorticopulmonary septum [55,56]. Defect components that are involved in induction, delamination, and migration of the cranial neural crest can affect craniofacial development [57]. Cranial neural crest cells can differentiate into mesenchymal cell types canonically associated with the mesoderm lineage (bone, cartilage, and smooth muscle), in addition to cell types typically derived from the ectodermal layer (neurons and glia) [58].

**Figure 3:**
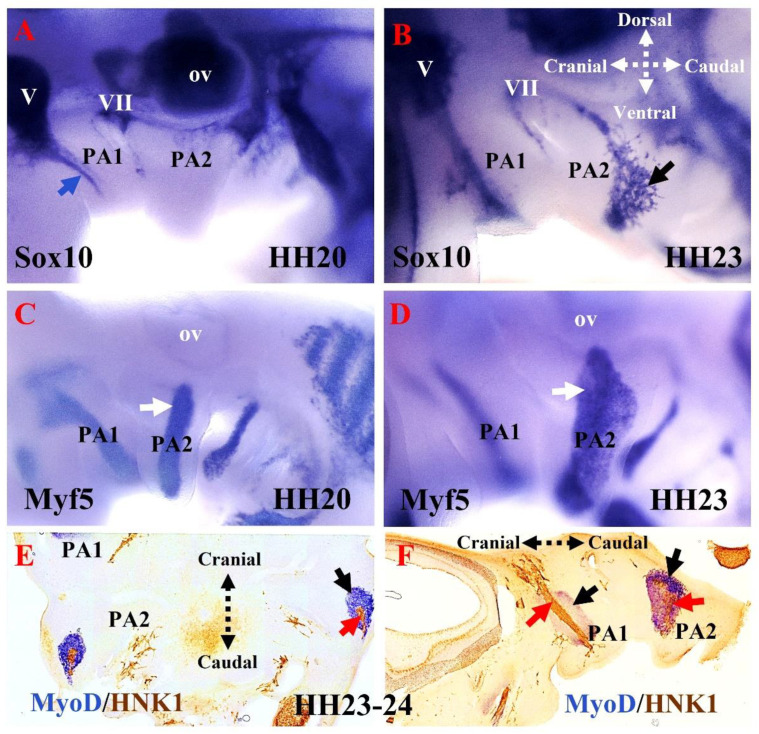
The emergence of the non-ectomesenchymal neural crest in the pharyngeal arches. (**A**,**B**) Analysis of Sox10 expression in developing chicken embryos using whole-mount in situ hybridization. Lateral views of the left side of chicken embryo stage HH20 and HH23. Sox10 was detected in the otic vesicle, facial ganglion, trigeminal ganglion, and first pharyngeal arch (blue arrow). Sox10 was first detected in the second pharyngeal arch at stage HH23 (facial nerve, black arrow). (**C**,**D**) Analysis of Myf5 expression using whole-mount in situ hybridization. (**E**) Frontal and sagittal (**F**) sections of a chicken embryo at the level of the second pharyngeal arch were hybridized with the MyoD probe, followed by immunostaining using an HNK1 antibody. Myf5 (white arrows) and MyoD (black arrow) mark the mesodermal core of the pharyngeal arches. In (**E**,**F**), cranial nerves in the first and second pharyngeal arches are revealed by HNK1 staining (red arrows). PA1, first pharyngeal arch; PA2, second pharyngeal arch; ov, otic vesicle; V, trigeminal ganglion; VII, facial ganglion.

**Figure 4:**
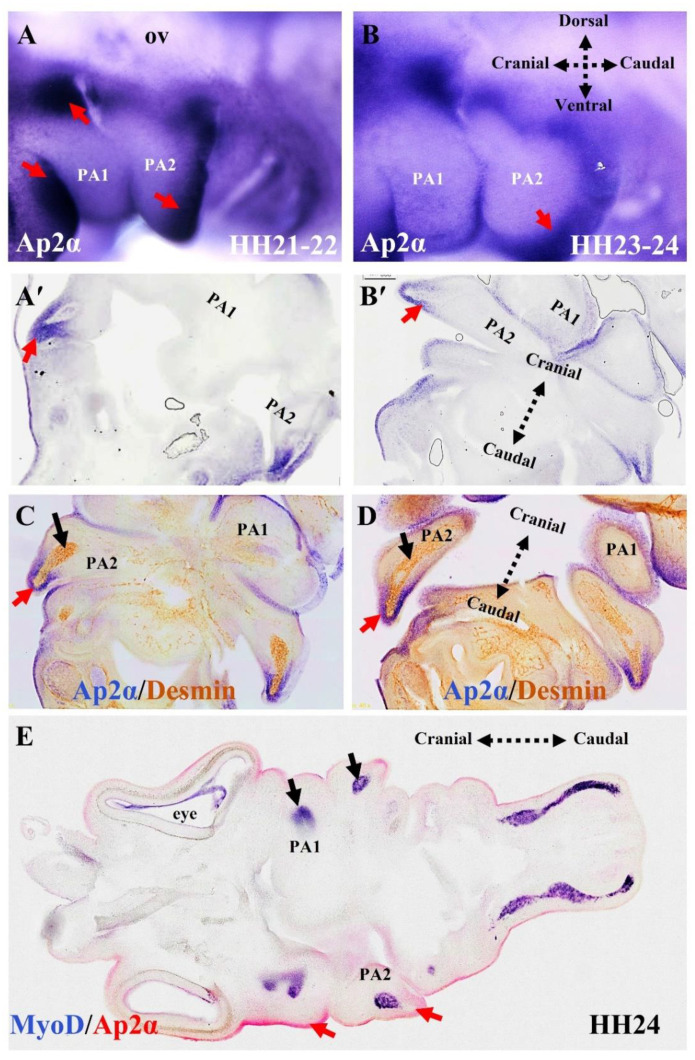
Ap2α marks ectomesenchymal neural crest cells in the first and second pharyngeal arches. (**A**,**B**) Analysis of Ap2α expression using whole-mount in situ hybridization. Lateral views of stages HH21-22 and HH23-24. (**A’**,**B’**) Adjacent vibratome frontal sections of the chicken embryos in (**A**,**B**) at the level of first and second pharyngeal arches. (**C**,**D**) Immunostaining for Desmin on the same frontal sections as in (**A’**,**B’**) after whole-mount in situ hybridization. The mesodermal core was visualized with Desmin antibody (black arrows). (**E**) The frontal section shows double whole-mount in situ hybridization for MyoD (blue) and Ap2α (red). Note that Ap2α marks the ectomesnchymal neural crest (red arrows) and MyoD marks the mesodermal core of the pharyngeal arches (black arrows). PA1, first pharyngeal arch; PA2, second pharyngeal arch; ov, otic vesicle.

The development of the cranial neural crest can be traced using a range of molecular markers [50]. In the chicken, the Sox10E2 enhancer is one of the earliest-acting neural crest cis-regulatory elements. It is critical for the onset of Sox10 expression in the newly formed cranial neural crest, but not the trunk and vagal neural crest [59]. As a non-ectomesenchymal neural crest cell marker, Sox10 is a key transcription factor involved in the early specification of multiple neural crest lineages (melanocytes, glia, and autonomic neurons) [59]. Sox10 expression is maintained in the migrating cranial neural crest cells that lie between the hindbrain and the pharyngeal arches (Figure 3A,B). These dorsally located neural crest cells contribute to the neurons and glia of the cranial ganglia (Figure 3A) [50]. At a later stage, the Sox10-expressing cells invade the mesenchyme of the second pharyngeal arch (Figure 3B). The transcription factor activating protein-2 alpha (Ap2α) is also expressed early in neural crest development and is implicated in face morphogenesis [60]; however, its expression also extends into the non-neural ectoderm [61]. The functions of Sox10 and Ap2α have been studied in chicken and mouse embryos [27,28,50,59,60,62,63,64,65]. The cranial neural crest cells that invade the pharyngeal arches (Figure 4) and give rise to the ectomesenchymal derivatives (cranial skeleton, cartilage, and connective tissue) of the head and neck [6,50,51,53,60,66] do not express Sox10, but rather express Ap2α [50]. These ectomesenchymal derivatives, which form part of many structures, including the jaws, are thought to be a key element at the center of vertebrate evolution and diversity [67]. The human natural killer-1 (HNK1) carbohydrate epitope is expressed in the neural crest cells and is involved in cell migration [52,68,69]. Considering neural crest morphology and migration details, HNK1 is often a better marker than Sox10 [52]. Thus, HNK1, Sox10, and Ap2α permit analysis of the respective location of neural crest cells in the pharyngeal arches [60]. Previously, using double in situ hybridization analysis of whole mount chicken embryos, we reported that Sox10 and Ap2α expression can be used to analyze the respective location of neural crest cells in the pharyngeal arches [27]. More recently, we have revealed that non-ectomesenchymal neural crest cell invasion of the second pharyngeal arch is delayed compared with that of the first pharyngeal arch in chicken embryos (Figure 4B) [70]. We also observed HNK1-positive nerve fibers invading the mesodermal core of the pharyngeal arches (Figure 3E,F). Cranial neural crest cells play an essential role in branchiomeric muscle differentiation and subsequent spatial organization [46]. Furthermore, it has been reported that neural crest cells regulate myogenesis in the head region by specifically influencing the rate of cell proliferation and differentiation [47].

## 6. Branchiomeric Muscles Are Heterogeneous in Terms of the Onset of Their Myogenic Differentiation

Muscle cell differentiation can be defined as a unidirectional process that progresses through a series of lineage-restraint events, with cellular multipotential being gradually reduced as embryonic development proceeds [58]. Cells undertaking myogenic differentiation switch on the expression of myogenic regulatory factor genes Myf5 and MyoD [10,11]. Once these genes are expressed, myogenic differentiation is thought to occur similarly in the head and trunk [2]. Myf5 and MyoD are expressed first and promote myoblast differentiation [2,14]. These transcription factors are important for activating genes that encode structural and contractile proteins that form the muscle fibers [71,72]. Thus, MyoD and Myf5 trigger the expression of genes for terminal differentiation [45,72,73].

In somites, activation of Myf5 and MyoD occurs first in the epaxial myotome, later in the hypaxial myotome, and lastly in migrating progenitor cells that enter limb buds [10]. Signals from the notochord and neural tube specifically are thought to promote the formation of the epaxial muscle anlagen, which remains near the axial midline tissues to form the intrinsic back muscles [11,74]. BMP signals have been reported to block myogenesis in both the trunk and head regions [11,49,75,76,77,78]. Notably, BMP inhibitors such as Noggin and Gremlin (Figure 2), as well as Wnt inhibitor signals (Frzb) secreted by both cranial neural crest cells and other tissues, were shown to induce myogenesis in branchiomeric muscles [11,48,49]. Terminal myogenic differentiation is marked by Myogenin (MyoG) expression [2,8], which promotes the differentiation of myoblasts into myotubes (contractile cells) [2,8,11,79]. Shortly after formation, the primary myotubes begin to express several myosin heavy chain (MyHC) genes. According to muscle type and developmental phase, each MyHC gene shows a specific pattern of expression [80]. Seven MyHC isoforms are organized in a cluster on syntenic regions of mouse chromosome 11 [81]. Five MyHC isoforms (MyHC-IIa, MyHC-IIx, MyHC-IIb, MyHC-slow, and MyHC-extraocular) are expressed in a mosaic pattern during adult life [72,80]. Only two MyHC isoforms (MyHC-embryonic and MyHC-perinatal) are transiently expressed during embryonic, fetal, and neonatal development [80]. These developmental MyHC isoforms disappear shortly after birth when adult MyHC isoforms become prevalent. However, developmental MyHC isoforms are re-expressed during muscle regeneration [82]. Therefore, the presence of these isoforms in the pathologic skeletal muscle indicates muscle fiber regeneration [82].

**Figure 5:**
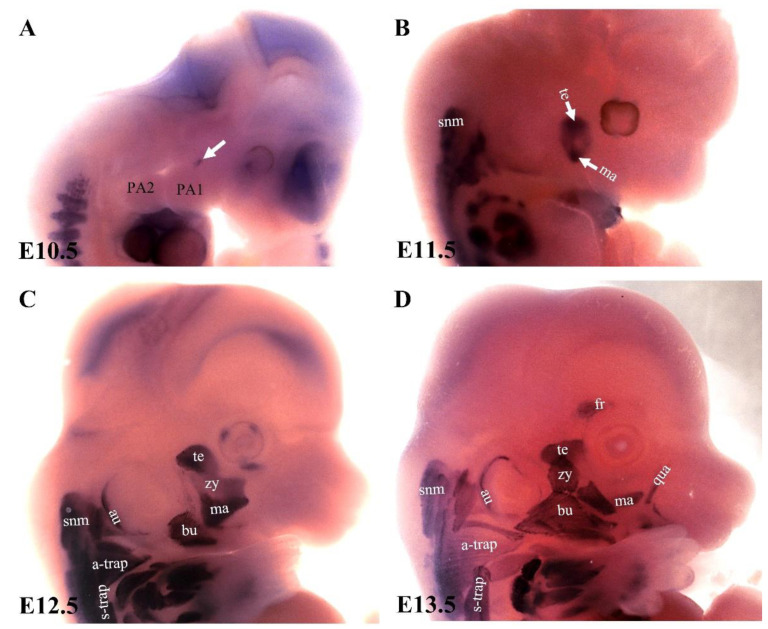
The emergence of myosin heavy chain during the development of the mouse branchiomeric muscle. (**A**–**D**) Analysis of myosin heavy chain (MyHC) expression using whole-mount in situ hybridization. Lateral views of E10.5, E11.5, E12.5, and E13.5 mouse embryos. The MyHC transcripts emerged in the first arch-derived muscle anlagen (white arrow in (**A**)) before the second and caudal brachial-derived muscle anlagen. Note: a-trap, acromiotrapezius; au, auricularis; bu; buccinator; fr, frontalis; ma, masseter; PA1, first pharyngeal arch; PA2, second pharyngeal arch; qu, quadratus labii; snm, somitic neck muscle; s-trap, spinotrapezius; te, temporalis, zy, zygomaticus.

The expression pattern of MyHC in the chicken embryo has previously been described [9,10]. In the chicken embryo, the primary myotubes in the head region turn on MyHC at stage HH32 [10]. Little is known about the emergence of the MyHC genes during the development of branchiomeric muscles in the mouse embryo. Therefore, an important open question in the field regards when MyHC expression emerges in branchiomeric muscle anlagen. We have recently addressed this question in mouse embryos by analyzing the onset of its expression in the branchiomeric muscles [70]. MyHC was first detected at embryonic day 10.5 (Figure 5A), and its peak expression occurred around embryonic day 13.5 (Figure 5D). Interestingly, muscle cells that originated from the mesodermal core of the first arch expressed MyHC rapidly and formed mastication muscles (Figure 5A). In contrast, myogenic cells, which originated from the second pharyngeal arch and formed caudal pharyngeal arches, delayed MyHC expression by approximately two days (Figure 5C). These delays were in accordance with the late invasion of Sox10-positive neural crest cells that populated the chicken’s second pharyngeal arch. These cells formed the sensory ganglia of the seventh cranial nerve that innervates myogenic cells in the mesodermal core (Figure 3B). It has been well documented that skeletal muscle growth during embryogenesis requires a fine balance between proliferation and differentiation [75]. It was proposed that Wnt and BMP signals play a role in the delayed differentiation of branchiomeric progenitor cells within regulatory circuits involving pre-myogenic specification factors such as Pitx2, Tbx1, and MyoR. Thus, these signals control the balance between myogenic progenitor cell proliferation and differentiation in the head [11]. It was recently reported that developmental MyHC isoforms persist throughout adult stages in the first pharyngeal arch-derived (mastication) muscles [82], but are absent in the second and most caudal arch-derived muscles. These findings supported the fact that the divergence between the myogenic programs of the first and second arch-derived muscle not only exists during development, but also maintains their respective embryonic regulatory signatures during adult life.

## 7. Conclusions

Here, we reviewed a new level of divergence within branchiomeric muscles. This diversity seems to be reflected by a tight relationship between the branchiomeric muscle progenitors and cranial neural crest cells. The degree to which branchiomeric muscle differentiation is regulated by signals secreted by cranial neural crest cells is a subject that requires further in-depth analysis. Furthermore, the cardiopharyngeal mesoderm delivers adult skeletal muscle stem cells that maintain some of their respective embryonic regulatory signatures. These diverse novel genetic programs of branchiomeric muscles may bring new insights into congenital disorders, which often affect only subsets of skeletal muscles. Further research in this area will provide critical information for characterizing the properties of the cardiopharyngeal mesoderm, and might be redirected toward the therapeutic potential for muscular diseases.

## Data Availability

All data generated during the current study are present in the paper. Additional data related to this paper may be requested from the corresponding authors (I.Y. or G.M.-P.).

## References

[1] Lescroart F., Kelly R.G., Le Garrec J.F., Nicolas J.F., Meilhac S.M., Buckingham M. (2010). Clonal analysis reveals common lineage relationships between head muscles and second heart field derivatives in the mouse embryo. Development.

[2] Nogueira J.M., Hawrot K., Sharpe C., Noble A., Wood W.M., Jorge E.C., Goldhamer D.J., Kardon G., Dietrich S. (2015). The emergence of Pax7-expressing muscle stem cells during vertebrate head muscle development. Front. Aging Neurosci..

[3] Buckingham M., Vincent S.D. (2009). Distinct and dynamic myogenic populations in the vertebrate embryo. Curr. Opin. Genet. Dev..

[4] Sambasivan R., Kuratani S., Tajbakhsh S. (2011). An eye on the head: The development and evolution of craniofacial muscles. Development.

[5] Buckingham M., Rigby P.W.J. (2014). Gene Regulatory Networks and Transcriptional Mechanisms that Control Myogenesis. Dev. Cell.

[6] Heude E., Tesarova M., Sefton E.M., Jullian E., Adachi N., Grimaldi A., Zikmund T., Kaiser J., Kardon G., Kelly R.G. (2018). Unique morphogenetic signatures define mammalian neck muscles and associated connective tissues. eLife.

[7] Lu J.R., Bassel-Duby R., Hawkins A., Chang P., Valdez R., Wu H., Gan L., Shelton J.M., Richardson J.A., Olson E.N. (2002). Control of facial muscle development by MyoR and capsulin. Science.

[8] Shih H.P., Gross M.K., Kioussi C. (2007). Cranial muscle defects of Pitx2 mutants result from specification defects in the first branchial arch. Proc. Natl. Acad. Sci. USA.

[9] Noden D.M., Francis-West P. (2006). The differentiation and morphogenesis of craniofacial muscles. Dev. Dyn..

[10] Noden D.M., Marcucio R., Borycki A.G., Emerson C.P. (1999). Differentiation of avian craniofacial muscles: I. Patterns of early regulatory gene expression and myosin heavy chain synthesis. Dev. Dyn. Off. Publ. Am. Assoc. Anat..

[11] Tzahor E., Brand-Saberi B. (2015). Head Muscle Development. Vertebrate Myogenesis: Stem Cells and Precursors.

[12] Buckingham M., Relaix F. (2015). PAX3 and PAX7 as upstream regulators of myogenesis. Semin. Cell Dev. Biol..

[13] Christ B., Ordahl C.P. (1995). Early stages of chick somite development. Anat. Embryol..

[14] Shih H.P., Gross M.K., Kioussi C. (2008). Muscle development: Forming the head and trunk muscles. Acta Histochem..

[15] Brent A.E., Schweitzer R., Tabin C.J. (2003). A somitic compartment of tendon progenitors. Cell.

[16] Parker M.H., Seale P., Rudnicki M.A. (2003). Looking back to the embryo: Defining transcriptional networks in adult myogenesis. Nat. Rev. Genet..

[17] Pu Q., Abduelmula A., Masyuk M., Theiss C., Swandulla D., Hans M., Patel K., Brand-Saberi B., Huang R.J. (2013). The dermomyotome ventrolateral lip is essential for the hypaxial myotome formation. BMC Dev. Biol..

[18] Dietrich S., Schubert F.R., Lumsden A. (1997). Control of dorsoventral pattern in the chick paraxial mesoderm. Development.

[19] Hollway G., Currie P. (2005). Vertebrate myotome development. Birth Defects Res. Part C Embryo Today Rev..

[20] Ordahl C.P., Berdougo E., Venters S.J., Denetclaw W. (2001). The dermomyotome dorsomedial lip drives growth and morphogenesis of both the primary myotome and dermomyotome epithelium. Development.

[21] Vasyutina E., Stebler J., Brand-Saberi B., Schulz S., Raz E., Birchmeier C. (2005). CXCR4 and Gab1 cooperate to control the development of migrating muscle progenitor cells. Genes Dev..

[22] Lours-Calet C., Alvares L.E., El-Hanfy A.S., Gandesha S., Walters E.H., Sobreira D.R., Wotton K.R., Jorge E.C., Lawson J.A., Lewis A.K. (2014). Evolutionarily conserved morphogenetic movements at the vertebrate head–trunk interface coordinate the transport and assembly of hypopharyngeal structures. Dev. Biol..

[23] Adachi N., Pascual-Anaya J., Hirai T., Higuchi S., Kuroda S., Kuratani S. (2018). Stepwise participation of HGF/MET signaling in the development of migratory muscle precursors during vertebrate evolution. Zool. Lett..

[24] Sefton E.M., Gallardo M., Kardon G. (2018). Developmental origin and morphogenesis of the diaphragm, an essential mammalian muscle. Dev. Biol..

[25] Comai G., Heude E., Mella S., Paisant S., Pala F., Gallardo M., Langa F., Kardon G., Gopalakrishnan S., Tajbakhsh S. (2019). A distinct cardiopharyngeal mesoderm genetic hierarchy establishes antero-posterior patterning of esophagus striated muscle. eLife.

[26] Lescroart F., Dumas C.E., Adachi N., Kelly R.G. (2022). Emergence of heart and branchiomeric muscles in cardiopharyngeal mesoderm. Exp. Cell Res..

[27] Yahya I., Boing M., Pu Q., Puchert M., Oedemis V., Engele J., Brand-Saberi B., Morosan-Puopolo G. (2020). Cxcr4 and Sdf-1 are critically involved in the formation of facial and non-somitic neck muscles. Sci. Rep..

[28] Yahya I., Morosan-Puopolo G., Brand-Saberi B. (2020). The CXCR4/SDF-1 Axis in the Development of Facial Expression and Non-somitic Neck Muscles. Front. Cell Dev. Biol..

[29] Tzahor E., Evans S.M. (2011). Pharyngeal mesoderm development during embryogenesis: Implications for both heart and head myogenesis. Cardiovasc. Res..

[30] Diogo R., Kelly R.G., Christiaen L., Levine M., Ziermann J.M., Molnar J.L., Noden D.M., Tzahor E. (2015). A new heart for a new head in vertebrate cardiopharyngeal evolution. Nature.

[31] Lescroart F., Hamou W., Francou A., Theveniau-Ruissy M., Kelly R.G., Buckingham M. (2015). Clonal analysis reveals a common origin between nonsomite-derived neck muscles and heart myocardium. Proc. Natl. Acad. Sci. USA.

[32] Wang X., Chen D., Chen K., Jubran A., Ramirez A., Astrof S. (2017). Endothelium in the pharyngeal arches 3, 4 and 6 is derived from the second heart field. Dev. Biol..

[33] Yahya I., Al Haj A., Brand-Saberi B., Morosan-Puopolo G. (2020). Chicken Second Branchial Arch Progenitor Cells Contribute to Heart Musculature in vitro and in vivo. Cells Tissues Organs.

[34] Kelly R.G., Brown N.A., Buckingham M.E. (2001). The arterial pole of the mouse heart forms from Fgf10-expressing cells in pharyngeal mesoderm. Dev. Cell.

[35] Gopalakrishnan S., Comai G., Sambasivan R., Francou A., Kelly R.G., Tajbakhsh S. (2015). A Cranial Mesoderm Origin for Esophagus Striated Muscles. Dev. Cell.

[36] Adachi N., Bilio M., Baldini A., Kelly R.G. (2020). Cardiopharyngeal mesoderm origins of musculoskeletal and connective tissues in the mammalian pharynx. Development.

[37] Bothe I., Tenin G., Oseni A., Dietrich S. (2011). Dynamic control of head mesoderm patterning. Development.

[38] Harel I., Nathan E., Tirosh-Finkel L., Zigdon H., Guimaraes-Camboa N., Evans S.M., Tzahor E. (2009). Distinct origins and genetic programs of head muscle satellite cells. Dev. Cell.

[39] Sambasivan R., Gayraud-Morel B., Dumas G., Cimper C., Paisant S., Kelly R.G., Tajbakhsh S. (2009). Distinct regulatory cascades govern extraocular and pharyngeal arch muscle progenitor cell fates. Dev. Cell.

[40] Gage P.J., Suh H., Camper S.A. (1999). Dosage requirement of Pitx2 for development of multiple organs. Development.

[41] Kitamura K., Miura H., Miyagawa-Tomita S., Yanazawa M., Katoh-Fukui Y., Suzuki R., Ohuchi H., Suehiro A., Motegi Y., Nakahara Y. (1999). Mouse Pitx2 deficiency leads to anomalies of the ventral body wall, heart, extra-and periocular mesoderm and right pulmonary isomerism. Development.

[42] Lu M.-F., Pressman C., Dyer R., Johnson R.L., Martin J.F. (1999). Function of Rieger syndrome gene in left–right asymmetry and craniofacial development. Nature.

[43] Kelly R.G., Jerome-Majewska L.A., Papaioannou V.E. (2004). The del22q11.2 candidate gene Tbx1 regulates branchiomeric myogenesis. Hum. Mol. Genet..

[44] Dong F., Sun X., Liu W., Ai D., Klysik E., Lu M.-F., Hadley J., Antoni L., Chen L., Baldini A. (2006). Pitx2 promotes development of splanchnic mesoderm-derived branchiomeric muscle. Development.

[45] Singh K., Dilworth F.J. (2013). Differential modulation of cell cycle progression distinguishes members of the myogenic regulatory factor family of transcription factors. FEBS J..

[46] Evans D.J., Noden D.M. (2006). Spatial relations between avian craniofacial neural crest and paraxial mesoderm cells. Dev. Dyn..

[47] Rinon A., Lazar S., Marshall H., Buchmann-Moller S., Neufeld A., Elhanany-Tamir H., Taketo M.M., Sommer L., Krumlauf R., Tzahor E. (2007). Cranial neural crest cells regulate head muscle patterning and differentiation during vertebrate embryogenesis. Development.

[48] Tirosh-Finkel L., Zeisel A., Brodt-Ivenshitz M., Shamai A., Yao Z., Seger R., Domany E., Tzahor E. (2010). BMP-mediated inhibition of FGF signaling promotes cardiomyocyte differentiation of anterior heart field progenitors. Development.

[49] Tzahor E., Kempf H., Mootoosamy R.C., Poon A.C., Abzhanov A., Tabin C.J., Dietrich S., Lassar A.B. (2003). Antagonists of Wnt and BMP signaling promote the formation of vertebrate head muscle. Genes Dev..

[50] Blentic A., Tandon P., Payton S., Walshe J., Carney T., Kelsh R.N., Mason I., Graham A. (2008). The emergence of ectomesenchyme. Dev. Dyn..

[51] Graham A. (2003). The neural crest. Curr. Biol..

[52] Giovannone D., Ortega B., Reyes M., El-Ghali N., Rabadi M., Sao S., de Bellard M.E. (2015). Chicken trunk neural crest migration visualized with HNK1. Acta Histochem..

[53] Minoux M., Rijli F.M. (2010). Molecular mechanisms of cranial neural crest cell migration and patterning in craniofacial development. Development.

[54] Maeda K., Asai R., Maruyama K., Kurihara Y., Nakanishi T., Kurihara H., Miyagawa-Tomita S. (2016). Postotic and preotic cranial neural crest cells differently contribute to thyroid development. Dev. Biol..

[55] Escot S., Blavet C., Hartle S., Duband J.L., Fournier-Thibault C. (2013). Misregulation of SDF1-CXCR4 signaling impairs early cardiac neural crest cell migration leading to conotruncal defects. Circ. Res..

[56] Kirby M.L., Gale T.F., Stewart D.E. (1983). Neural crest cells contribute to normal aorticopulmonary septation. Science.

[57] Siismets E.M., Hatch N.E. (2020). Cranial neural crest cells and their role in the pathogenesis of craniofacial anomalies and coronal craniosynostosis. J. Dev. Biol..

[58] Zalc A., Sinha R., Gulati G.S., Wesche D.J., Daszczuk P., Swigut T., Weissman I.L., Wysocka J. (2021). Reactivation of the pluripotency program precedes formation of the cranial neural crest. Science.

[59] Betancur P., Bronner-Fraser M., Sauka-Spengler T. (2010). Genomic code for Sox10 activation reveals a key regulatory enhancer for cranial neural crest. Proc. Natl. Acad. Sci. USA.

[60] Grenier J., Teillet M.A., Grifone R., Kelly R.G., Duprez D. (2009). Relationship between Neural Crest Cells and Cranial Mesoderm during Head Muscle Development. PLoS ONE.

[61] Huang X., Saint-Jeannet J.-P. (2004). Induction of the neural crest and the opportunities of life on the edge. Dev. Biol..

[62] Yahya I., Böing M., Brand-Saberi B., Morosan-Puopolo G. (2021). How to distinguish between different cell lineages sharing common markers using combinations of double in-situ-hybridization and immunostaining in avian embryos: CXCR4-positive mesodermal and neural crest-derived cells. Histochem. Cell Biol..

[63] Pusch C., Hustert E., Pfeifer D., Südbeck P., Kist R., Roe B., Wang Z., Balling R., Blin N., Scherer G. (1998). The SOX10/Sox10 gene from human and mouse: Sequence, expression, and transactivation by the encoded HMG domain transcription factor. Hum. Genet..

[64] Cheng Y.-C., Cheung M., Abu-Elmagd M.M., Orme A., Scotting P.J. (2000). Chick sox10, a transcription factor expressed in both early neural crest cells and central nervous system. Dev. Brain Res..

[65] Buac K., Watkins-Chow D.E., Loftus S.K., Larson D.M., Incao A., Gibney G., Pavan W.J. (2008). A Sox10 expression screen identifies an amino acid essential for Erbb3 function. PLoS Genet..

[66] Fabian P., Tseng K.-C., Thiruppathy M., Arata C., Chen H.-J., Smeeton J., Nelson N., Crump J.G. (2022). Lifelong single-cell profiling of cranial neural crest diversification in zebrafish. Nat. Commun..

[67] Prasad M.S., Charney R.M., García-Castro M.I. (2019). Specification and formation of the neural crest: Perspectives on lineage segregation. Genesis.

[68] Betters E., Charney R.M., Garcia-Castro M.I. (2018). Early specification and development of rabbit neural crest cells. Dev. Biol..

[69] Yagi H., Yanagisawa M., Suzuki Y., Nakatani Y., Ariga T., Kato K., Robert K.Y. (2010). HNK-1 epitope-carrying tenascin-C spliced variant regulates the proliferation of mouse embryonic neural stem cells. J. Biol. Chem..

[70] Yahya I., Böing M., Hockman D., Brand-Saberi B., Morosan-Puopolo G. (2022). The Emergence of Embryonic Myosin Heavy Chain during Branchiomeric Muscle Development. Life.

[71] Agarwal M., Sharma A., Kumar P., Kumar A., Bharadwaj A., Saini M., Kardon G., Mathew S.J. (2020). Myosin heavy chain-embryonic regulates skeletal muscle differentiation during mammalian development. Development.

[72] Schubert F.R., Singh A.J., Afoyalan O., Kioussi C., Dietrich S. (2018). To roll the eyes and snap a bite–function, development and evolution of craniofacial muscles. Semin. Cell Dev. Biol..

[73] Kitzmann M., Fernandez A. (2001). Crosstalk between cell cycle regulators and the myogenic factor MyoD in skeletal myoblasts. Cell. Mol. Life Sci. CMLS.

[74] Burke A.C., Nowicki J. (2003). A new view of patterning domains in the vertebrate mesoderm. Dev. Cell.

[75] Amthor H., Christ B., Patel K. (1999). A molecular mechanism enabling continuous embryonic muscle growth-a balance between proliferation and differentiation. Development.

[76] McMahon J.A., Takada S., Zimmerman L.B., Fan C.-M., Harland R.M., McMahon A.P. (1998). Noggin-mediated antagonism of BMP signaling is required for growth and patterning of the neural tube and somite. Genes Dev..

[77] Tirosh-Finkel L., Elhanany H., Rinon A., Tzahor E. (2006). Mesoderm progenitor cells of common origin contribute to the head musculature and the cardiac outflow tract. Development.

[78] Von Scheven G., Alvares L.E., Mootoosamy R.C., Dietrich S. (2006). Neural tube derived signals and Fgf8 act antagonistically to specify eye versus mandibular arch muscles. Development.

[79] Penn B.H., Bergstrom D.A., Dilworth F.J., Bengal E., Tapscott S.J. (2004). A MyoD-generated feed-forward circuit temporally patterns gene expression during skeletal muscle differentiation. Genes Dev..

[80] Beylkin D.H., Allen D.L., Leinwand L.A. (2006). MyoD, Myf5, and the calcineurin pathway activate the developmental myosin heavy chain genes. Dev. Biol..

[81] Weiss A., McDonough D., Wertman B., Acakpo-Satchivi L., Montgomery K., Kucherlapati R., Leinwand L., Krauter K. (1999). Organization of human and mouse skeletal myosin heavy chain gene clusters is highly conserved. Proc. Natl. Acad. Sci. USA.

[82] Schiaffino S., Rossi A.C., Smerdu V., Leinwand L.A., Reggiani C. (2015). Developmental myosins: Expression patterns and functional significance. Skelet Muscle.

